# A Study on Mechanical and Microstructural Characteristics of Concrete Using Recycled Aggregate

**DOI:** 10.3390/ma15217535

**Published:** 2022-10-27

**Authors:** Herbert Sinduja Joseph, Thamilselvi Pachiappan, Siva Avudaiappan, Erick I. Saavedra Flores

**Affiliations:** 1 Division of Structural Engineering, Department of Civil Engineering, College of Engineering Guindy, Anna University, Chennai 600025, India; 2 Departamento de Ingeniería Civil, Universidad de Concepción, Concepción 4070386, Chile; 3 Centro Nacional de Excelencia para la Industria de la Madera (CENAMAD), Pontificia Universidad Católica de Chile, Av. Vicuña Mackenna, Santiago 4860, Chile; 4 Departamento de Ingeniería en Obras Civiles, Universidad de Santiago de Chile, Av. Ecuador, Santiago 3659, Chile

**Keywords:** mixed coarse recycled aggregate, sustainability, construction and demolition waste, mechanical properties, microstructural studies

## Abstract

The objective of this paper is to provide a comprehensive study about the performance of concrete using mixed coarse recycled aggregate (MCRA) as an alternative for natural aggregate (NA) at replacement levels of 0, 30, 60, and 100%, which can greatly reduce the environmental pollution by incorporating the construction and demolition wastes in the reproduction of concrete. The focus of this study was to use the raw MCRA that was directly obtained from a recycling plant and not further processed. Initially, MCRA was studied to ascertain if its property meets the recommended Indian standards for natural aggregates. Using the slump test, the workability of freshly prepared concrete with a characteristic strength of 30 MPa was assessed. Additionally, the mechanical performance of concrete was assessed on the specimens prepared in the different forms: cubes, cylinders, and beams. Moreover, Scanning Electron Microscopy (SEM) with EDAX, XRD, and FTIR were used to study the microstructural behavior of selected optimum and control mixes at 7 and 28 days of curing. The studies revealed that a higher MCRA content improved the workability of concrete and 30% replacement of MCRA improved the compressive strength by 11.01, 6.98, 6.19, and 14.24% at 7, 28, 56, and 90 days respectively. At the same time, the 30% replacement of the MCRA mix showed an improved split tensile and flexural strength by 2.92 and 6.26%, respectively. The microstructural analysis showed that the optimum mixture had a more condensed microstructure. Therefore, 30% replacement of MCRA can be incorporated in the characteristic strength of concrete of 30 MPa. In particular, MCRA incorporation had a positive influence similar to conventional concrete on the physical, mechanical, and microstructural properties, which can increase the utilization of all kinds of directly obtained construction and demolition wastes to increase the circular economy in the construction sector.

## 1. Introduction

Concrete is a human-made substance that is the most widely used material on the planet. At the same time, unfortunately, it represents a source of concern when it comes to environmental degradation. It is of maximum importance to investigate the materials used to manufacture concrete, as well as their broader environmental impact. It is expected that global annual construction waste could reach 2.2 billion tons by 2025 as reported by transparency market research. Based on the report of the building materials and technology promotion council [1], India produces approximately 150 million tons of Construction and Demolition Waste (C&DW) every year. As the rate of demolition of old structures has increased, enormous amounts of C&DW debris have been generated [2,3]. In most countries, C&DWs are frequently disposed of in landfills, leading to landfill space depletion. The generation of such a large amount of C&DW without proper management will have negative consequences for the environment, including water pollution and greenhouse gas emissions. As a result, there is a pressing need to dispose of and reclaim C&DW in a timely manner [4,5,6].

The crushed C&DW particles are mainly composed of concrete, mortar, bricks, glass, and wood pieces and can be used as mixed coarse recycled aggregate (MCRA) [7,8,9]. The physical properties of MCRA are complex owing to its various components. The recycled aggregates in different regions may have significant differences in their components. The complex components of MCRA lower its density [10] and flakiness index [11] and increase its water absorption [12] and crushing index [13]. Thus, it is a challenge to reuse MCRA efficiently as a conventional aggregate [14]. Still, the author reported that of the concretes containing less than 50% of mixed recycled coarse aggregates, both the compressive and flexure strength do not vary considerably. The morphological analysis showed that the recycled aggregate and natural aggregate interfacial transition zone (ITZ) are very similar [15,16]. An earlier study conducted on coarse (CMRA) and fine (FMRA) mixed recycled aggregate obtained from the C&DW management plant located in Caceres (Spain) found that the physical and mechanical requirements meet the EN 933-11 regulation [17]. 

The inclusion of MCRA at 25 and 50% replacement causes no consequential changes in the 28-day strength with respect to the standard mix. The tensile strength of concrete with 25 and 50% substitution of MCRA diminishes with 3 and 10%, progressively, in succession to the control mix [18]. This encouraging research has cleared the path for more efficient utilization of construction and demolition waste [19,20], as well as the durability study on the permeability of aggressive agents through the permeability of oxygen and chloride ions and the resistance to carbonation in concrete with 10 or 25% ground recycled cement (GRC) and 50% MCRA. The reinforcement’s passivity is not compromised by using up to 10% GRC as a cement replacement and/or 50% MCRA as a NA substitute in accordance with the service life prediction model proposed in the Spanish code of EHE-08 [14]. Most of the researchers enhanced the inferior properties of MCRA through various treatments such as CO_2_ curing [21], carbonation [22], acid treatment [23], heating [24], heating and scrubbing [25], the combination of mechanical, chemical, and thermal treatment [26], and the addition of various supplementary cementitious materials [27,28,29,30]. Treated mixed recycled aggregates have a lot of promise for use in structural applications, which are not subject to relentless environmental conditions [31].

The main objective of this research is to advance the adaptation of new types of aggregate in the form of a mixed coarse recycled aggregate fraction of building and demolition debris. Advancing research on recycled aggregate concrete, recycled aggregate collected from the C&DW recycling facility is used to produce recycled aggregate that has resulted in demolition waste utilization more broadly in the construction sector. However, examination of such recycled aggregate from the C&DW reprocessing plants is still required for the mechanical characteristics and microstructural behavior of concrete. The previous study’s hindrance has been overcome using mixed recycled aggregate, and the mechanical properties and microstructural characterization have been investigated deeply at a curing period of 7 and 28 days.

## 2. Experimental Program

The experimental study incorporated concrete with a varied percentage of recycled aggregates at 0, 30, 60, and 100 to determine the effect of recycled aggregate concrete at low, moderate, and high volumes. In this research, the mechanical properties of mixed recycled aggregate concrete such as compressive, flexure, and split tensile strength were investigated. Furthermore, nondestructive parameters of selected concrete series were evaluated in terms of the ultrasonic pulse velocity and rebound hammer. The morphology and cement hydration products of the optimum and control mix were studied using Scanning Electron Microscopy with Energy-Dispersive X-ray Spectroscopy (SEM-EDAX), X-ray Diffraction (XRD), and Fourier Transform Infrared Radiation (FT-IR).

### 2.1. Materials

In this current research, mixed coarse recycled aggregate was used as an alternative to conventional aggregate, which can increase the incorporation of all kinds of construction and demolition waste in concrete production. The natural aggregates utilized locally from Chennai, India and mixed recycled aggregates were sourced from the C&DW Recycling Plant at Jeedimetla in Telangana, India. The manufactured sand with a size less than 4.75 mm that belongs to Zone I as per IS383:2016 was used in this research. During the production of the reprocessed aggregates, the unsettled substances were discarded first from C&DWs and, thereafter, the construction waste was crushed by a jaw crusher and separated according to their sizes. The recycled aggregates involved in this study contained all forms of construction and demolition wastes except steel and floating materials. The coarse mixed recycled aggregates of 20 mm and 12 mm were mixed at a percentage of 60% and 40%, respectively, to enhance the properties of recycled aggregate concrete with better packing. Ordinary portland cement (53 grade) manufactured by Zuari, meeting IS 12269:2013 requirements, along with tap water was used for the experimental process. The superplasticizer (SP) with trading name AURAMIX 200 was used with a solid content of 30–35% having a specific gravity 1.08, which has a 20% water reduction rate. The amount of SP was found on the basis of workability requirements. 

**Figure 1:**
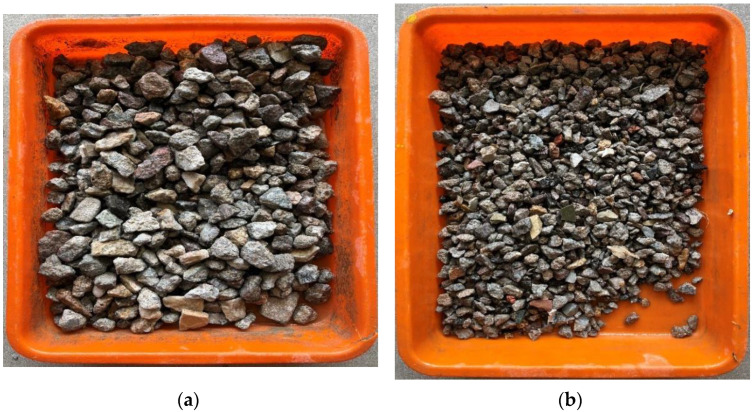
Coarse aggregates: (**a**) mixed coarse recycled aggregate (20 mm maximum size); (**b**) mixed coarse recycled aggregates (12 mm maximum size).

The coarse recycled material used in this study is shown in Figure 1. The typical composition of the recycled aggregate sample that was collected from the C&DW recycling plant at Jeedimetla, India was 73.8% fully reclaimed aggregate, 16% aggregate with partially adhered mortar, 4.45% tiles, 1.07% bricks, 1.63% black stones, 2.47% granite, 1.08% mosaic, and 1.97% others.

### 2.2. Physical Properties Studied

The physical properties of the aggregates obtained from the construction and demolition recycling plant at Jeedimetla were studied and the results are shown in Table 1. From the results, it can be observed that the mixed coarse recycled aggregates (MCRA) exhibited an almost similar nature compared to natural aggregates (NA). However, the water absorption of the MCRA was much higher than that of the NA. The 24 h water absorptions of MCRA of sizes 20 mm and 12 mm were 2.3% and 1.95%, which were higher than those of NA. Table 1 shows the water absorption of recycled aggregates in the present study, which is the same as those of the studies accomplished by various authors described in Table 2. The bulk density of MCRA was reduced at a percentage of 10.58% and 18.38% for 20 mm and 10 mm, respectively, compared with NA. Recycled aggregates showed a density reduction and a notably higher water absorption than NA. In addition, the aggregate crushing and impact value was almost twice the value of NA. Even though the physical properties differed when compared with natural aggregate, the strength properties were not affected significantly for recycled aggregate concrete [32]. This was because of the bond strength as the roughness of the aggregate increases [33]. However, the specific gravity of those recycled aggregates was similar to those of natural aggregates varying from 2.1 to 3.2 and satisfied the permissible limits IS 383:2016.

### 2.3. Granulometric Distribution of Aggregates (NA and MCRA)

**Figure 2:**
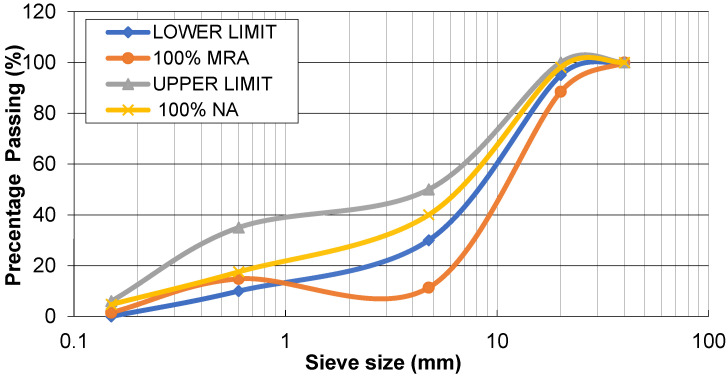
Grain size distribution for all in aggregates.

The particle size distributions of aggregates were determined using a sieve analysis test as per IS 383:2016. Figure 2 shows the various size ranges of all aggregates (both fine and coarse aggregates) for the 100% natural and mixed coarse recycled aggregate (MCRA) concrete mix compared with the upper and lower limits specified by IS 383:2016. The cumulative passing of all aggregates (both coarse and fine) for 100% natural and MCRA in the corresponding sieve size of 40, 20, 4.75, 0.6, and 0.15 mm was 100, 98.09, 40, 17.56, and 4.72% and 100, 88.45, 11.34, 14.80, and 1.52%, respectively. For the 100% MCRA curve, the gradation value deviated from the upper and lower limits in the sieve size of 4.75 mm at 11.35%. In addition, the deviation in the sieve size of 20 mm was 6.9%, which almost coincided with the lower limits. Despite the fact that the gradation differed from the control and did not meet the limits of grading requirements, the gradation was similar, indicating consistent RA generation. In addition, we chose to use the RA to make concrete without changing the gradations, which was supplied from the C&DW recycling plant. The choice was made to save expenditure and to make use of available gradations to achieve an acceptable particle distribution.

### 2.4. Mix Design

Four mixes were composed to explain the concrete in the fresh state, the hardened properties, and the microstructural behavior of concrete. The specimens were prepared in the different combinations: control mix with 100% NA; concrete with 30% MCRA (M1); concrete with 60% MCRA (M2); concrete with 100% MCRA (M3). The proportion of the mixes was designed using IS 10262:2019 shown in Table 3. All the mixes were designed to meet severe environmental conditions with an amount of binder of 380 kg/m^3^ and a water–cement ratio of 0.42 as per IS 456:2000. The mixes studied were prepared using a 45 L capacity laboratory vertical shaft mixer. All the dry ingredients were loaded one by one and dry-mixed for a duration of 120 s, and then water was added while the mixer was running; simultaneously, the superplasticizers were added to improve the workability. The total mixing time was 240 s, which satisfied the minimum requirement as per IS 456:2000. Finally, the samples were produced and cured by following IS 456:2000.

## 3. Results and Discussion

**Figure 3:**
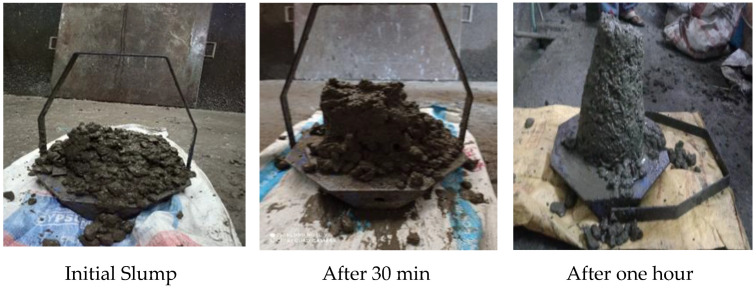
Concrete slump test.

**Figure 4:**
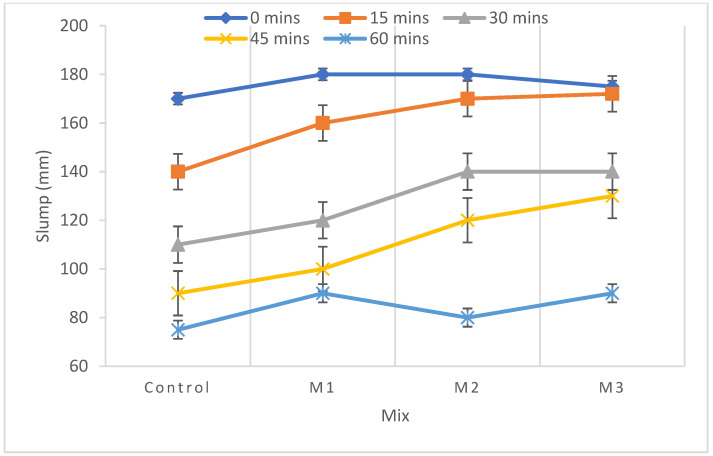
Variation in workability.

Fresh concrete properties, namely slump and density, were determined as specified in IS 456:2000. All the mixes were planned to have a retention slump value of one hour by fixing the dosage of superplasticizer (SP) ARAMIX 200 at 0.4% in the amount of binder. The variation in slump is shown in Figure 3. The slump loss range of all the mixes at different time intervals 0, 15, 30, 45, and 60 min is shown in Figure 4. In the graph of Figure 4, the mixes with recycled aggregates show higher slump values than the control mix due to more water absorption, which, in turn, increases the free water available for hydration [38]. All the mixes show an initial collapse with 0.4% of SP and, after one hour, all the mixes have a slump value around 75 ± 20 mm due to the absorption nature of recycled aggregates. The fresh density of all the mixes is nearer to that of conventional concrete, as shown in Table 4. Even the mix with 100% RA has a fresh density of 98% compared to that of conventional concrete. From these obtained results, it is worth noting that the fresh density is within the range of 2430–2300 kg/m^3^ for concrete that has different replacements of RA. This finding denotes that the tiles, mosaic, granite, and other components present in RA do not compromise the density of freshly prepared concrete. A similar study reported that the compressive strength of 25 MPa concrete showed a fresh density of 2416 and 2331.01 kg/m^3^ for ordinary concrete and 50% replacement of mixed recycled aggregate concrete (MRAC), respectively. The previous studies [16] found that the bulk density for the fresh concretes was 2.45 Mg/m^3^ for conventional and 2.28 Mg/m^3^ for 100% replacement of MRAC. Thus, it was found that the fresh concrete replaced with recycled aggregate has suitable properties for application of concrete.

### 3.1. Hardened Concrete Properties

#### 3.1.1. Compressive Strength

The hardened density values shown in Table 5 vary from 2532.74 to 2461.04 kg/m^3^ for the mix having NA and MRAC. In comparison with the control mix, the dry density of 30, 60, and 100% replacements of recycled aggregates decreases by 2.83, 2.78, and 1.6% respectively. This decrease in density is closer to the previous study by [17], which reported that the dry density of hardened concrete was 2416.37 and 2331.01 kg/m^3^ for the control and 50% replaced MRAC, respectively. This indicates that there was a 3.5% reduction in apparent dry density of MRAC while comparing with conventional concrete. The results of the current study show that the decrease in dry density of MRAC is less than the results obtained in previous studies.

**Figure 5:**
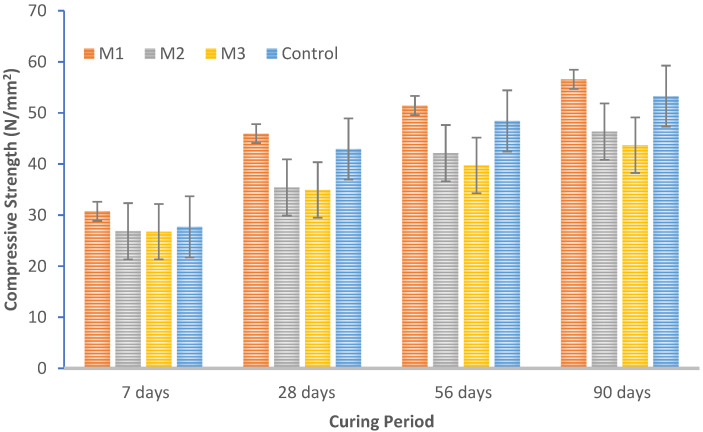
Variation in compressive strength.

Figure 5 demonstrates how the compressive strength of each recycled aggregate concrete mix increases over time in a manner comparable to the behavior of reference concrete. As shown in Figure 5, the compressive strength of recycled aggregate decreases beyond 30% replacement of MCRA, which can be attributed to the alteration in the water–cement ratio of the mix due to the higher water absorption of recycled aggregates during concreting. From Table 5, it can be observed that at 7, 28, 56, and 90 days, the strength of group M1 increases by 11.01, 6.98, 6.19, and 14.23% compared with the control mix. The relative compressive strength of all mix groups after seven days is in the range of 64.47–76.6% as that obtained after 28 days. At the 28th day, all the mixes satisfy the design strength of 30 MPa. The resulting design strength of MRAC was also achieved by an earlier study, which stated that even when the natural aggregate replacement percentage exceeded 80%, the mechanical performance of the recycled concrete generated with coarse recycled aggregates was comparable to that of regular concrete [39]. Further, at the 56th day, the strength increases at a percentage of 11.95, 19.01, 13.75, and 12.78% for M1, M2, M3, and the control mix, respectively, when compared to the 28th day’s compressive strength of each mix. Similarly, the 90th day strength increases at a percentage of 12.94, 10.7, 6.59, and 4.99% for M1, M2, M3, and the control mix, respectively, when compared to the 56th day’s compressive strength of each mix. However, compared with the control mix, it is observed that the compressive strength gradually decreases with increasing replacement percentage of MCRA in the concrete mixes. The reduced compressive strength obtained in the MRAC is because of the fact that recycled aggregate has different heterogenous components in the aggregate portion. As a result, the strength of concrete made with recycled aggregate is generally decreased by the interaction between the old mortar and fresh cement paste. Therefore, it is necessary to address the complex interaction behavior of recycled aggregate concrete. However, all of the mixes meet the design strength requirements on all curing days. Similar observations have been reported in previous studies by [17] who reported that after 28 days, the average strength was more than the 25 MPa design strength. As a result, these new recycled concretes might be utilized as structural concrete. Previous studies have demonstrated similar patterns in compressive strength gains across all age groups, regardless of the proportion of natural coarse aggregate replaced by recycled concrete aggregate. The same trend can be observed with the use of mixed recycled aggregate in this current study. Based on these observations, we conclude that the MCRA can be effectively used in structural applications. 

**Table 5:** Hardened concrete density and compressive strength.

SI. No	Mix ID	CS (N/mm^2^)	$∆\;f_{c}(\%)\;(♣)$	CS (N/mm^2^)	$∆\;f_{c}\left(\%\right)\;(♣)$	CS (N/mm^2^)	$∆\;f_{c}\left(\%\right)\;(♣)$	CS (N/mm^2^)	$∆\;f_{c}\left(\%\right)\;(♣)$	Dry Density (kg/m^3^)
		(7 Days)		(28 Days)		(56 Days)		(90 Days)		
1	M1	30.74	+11.01	45.95	+6.98	51.44	+6.19	58.10	+14.23	2461.04
2	M2	26.84	−3.07	35.40	−17.58	42.13	−13.03	46.64	−8.3	2462.22
3	M3	26.75	−3.4	34.92	−18.7	39.72	−18	42.34	−16.75	2492.15
4	control	27.69	-	42.95	-	48.44	-	50.86	-	2532.74

♣ Strength variation compared to control mix.

#### 3.1.2. Split Tensile and Flexural Strength

The bending and split tensile strength tests were carried out without applying any shock, and the load was gradually increased at a nominal rate between 1.2 and 2.4 N/mm^2^/min. The differences in strength compared to the control mix are shown in Table 6 along with the split tensile and flexure strengths of concrete tested after 28 days. From the results tabulated, it is evident that the higher amount of recycled aggregate in concrete results in a decrease in the strength of the interfacial transmission zone (ITZ). The tensile strength of MRAC is often lower than that of natural aggregate concrete. The extent of the difference depends on a number of variables, including the percentage of recycled aggregate replacement, water-reducing admixtures, and the impact of crushing and mixing processes [40]. Furthermore, from the obtained results, the concrete mix having 30% MCRA shows slightly better performance than the control mix both in flexural and split tensile strength. Similarly, in a previous study, it was found that the tensile strength of concrete produced with recycled coarse aggregate was nearly identical to that of concrete prepared with natural coarse aggregate [41]. In this study, for the concrete having coarse recycled aggregate with 100% replacement, the splitting tensile strength is reduced to 19%. Similar findings were reported with a 18% reduction in splitting tensile strength [42]. This reduction in tensile and split tensile strength of concrete is because of its various components, size, type, and quality. Moreover, the splitting tensile and flexure strength tests on specimens with recycled aggregate shows that the concrete frequently fails through the paste around the recycled aggregate.

#### 3.1.3. Nondestructive Testing

**Figure 6:**
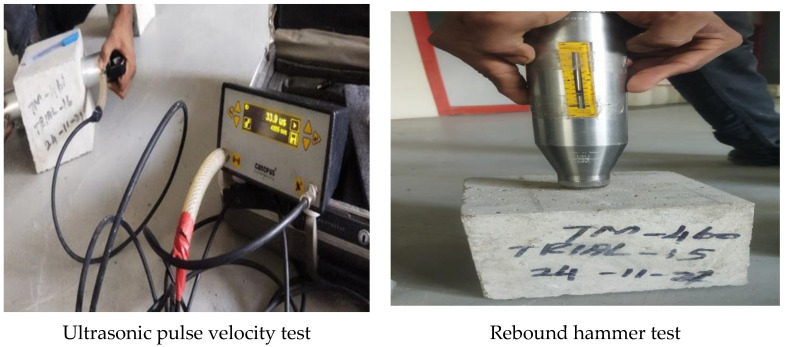
Nondestructive test setup.

In the present study, nondestructive testing of concrete was assessed as per IS 516 (part 5): 2018. Figure 6 shows the typical arrangement of the test setup for the ultrasonic pulse velocity (UPV) and rebound hammer tests. As shown in Figure 6, two transducers were placed on either side of the concrete specimen in order to assess the homogeneity of concrete specimens. The ultrasonic waves were allowed to pass between the two ends of the transducer to measure the UPV value in terms of m/s. As the side of the cube was 150 mm, the natural frequency of the transducer used here was 150 kHz as per the IS standards. In Table 7, it is seen that at 30% MCRA, the UPV is found to be 4495 m/s, and it slightly increases to 4655 m/s with concrete with 100% NA, i.e., control mix. Similar findings from an earlier study indicated that the reduction in pulse velocity caused by 50% replacement of RCAs in the mix was quite low, i.e., a small drop of up to 6% [43]. The very small decrease in UPV of MRAC at 30% replacement in the current study shows that the recycled aggregate replacement does not alter the phases of calcium silicate hydrate (CSH) in the pores by pozzolanic reaction. The blending of 20 mm and 12.5 mm at 60 and 40% coarse aggregates contributes to the increase in packing density of the cube with minimum voids, leading to better performance.

Figure 6 shows the Schmidt rebound hammer test to measure the surface hardness of the concrete specimens. Table 7 shows the Schmidt rebound hammer value of the specimen made with 30% replacement of MCRA and 100% natural aggregate. The 28-day rebound numbers of concrete with 30% incorporation of MCRA and the control mix are obtained as 32 and 36, respectively. The results of the current study are found to be higher than those of an earlier study that reported the 28-day rebound hammer value of concrete mix with 50% incorporation of RCA and control mix to be 26.4 and 23.9, respectively [44]. The lesser difference between the optimum and control mixes in the current study is due to the porous nature of MCRA that results in penetration of the cement paste into it than ordinary concretes.

### 3.2. Scanning Electron Microscopy with Energy-Dispersive Spectroscopy

**Figure 7:**
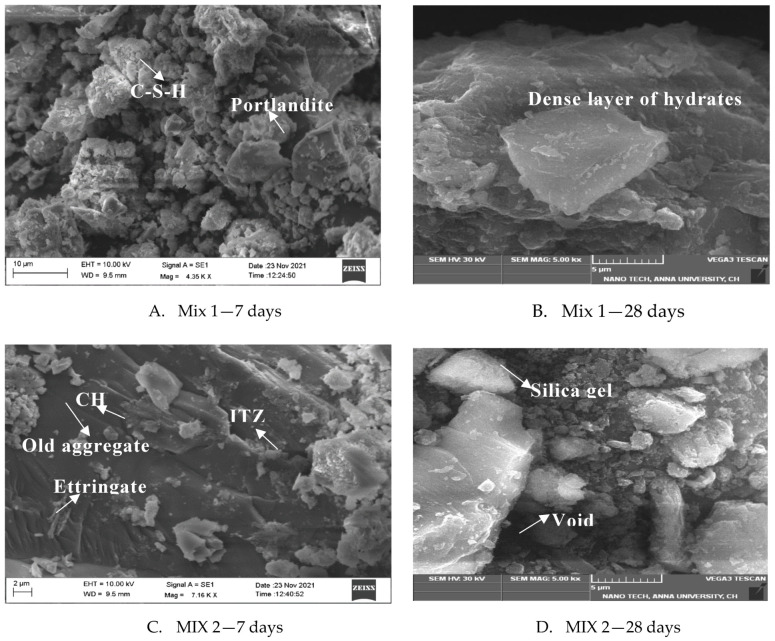

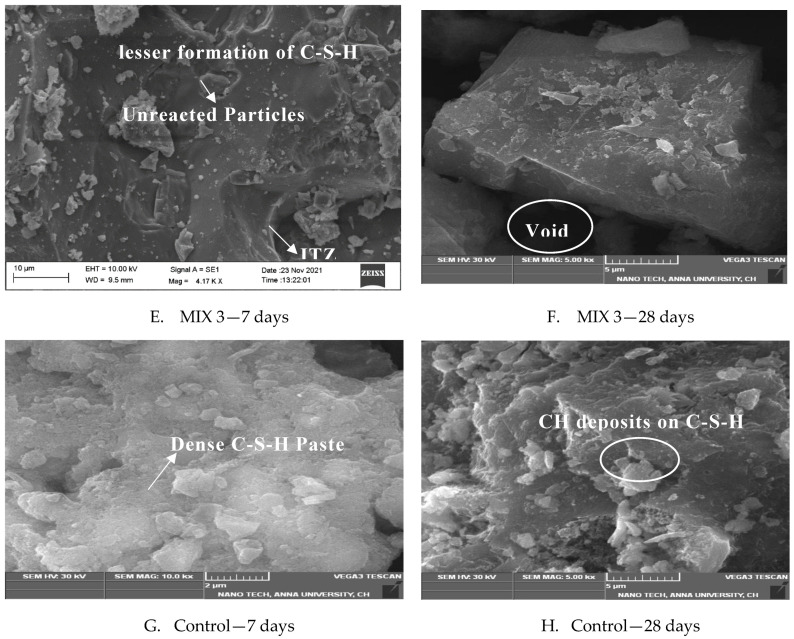
Secondary electron image using SEM.

The morphological characteristics of concrete mixtures were determined on the powder sample of a broken cube for both 7 and 28 days of curing. The TESCAN VEGA3 instrument manufactured from Brno, Czech Republic is used for scanning electron microscopy testing. All the absorbed water in the specimen was removed by hydration stoppage using the oven-drying method at 100 °C for a duration of 10 min. From Figure 7A, at 30% replacement of recycled aggregate concrete, the SEM observation shows that a dense ITZ is present in the layer between the recycled aggregate and the cement matrix. A strong layer of bond between the new cement paste and the aggregate is formed due to the rough surface and high porosity of MCRA, allowing water and cement paste to permeate the aggregate structure. The increase in surface tension results in a stronger bond between recycled coarse aggregate and cement matrix [45]. The increase in adhesion between cement paste and recycled coarse aggregate is due to the formation of hydration product deposits such as calcium silicate hydrate, portlandite, and calcite around the aggregates at such early times as 7 days, which is clearly seen at 30% replacement of MRAC. This is evident from Figure 7A where we see the presence of the large number of these small hydrated crystallites. Due to the creation of massive clusters of hydrated crystals, the voids and pores are filled completely. From Figure 7, we can observe that there is only limited ettringite formation, which is generally responsible for cracks in hardened concrete due to its expansion nature during the hydration process. As the hydration time increases up to 28 days, concrete mix densifies substantially. When compared with the control mix, there is no significant difference at 30% replacement of MCRA. An earlier, similar observation was that there was a stronger and denser new interfacial transition zone with fully replaced coarse recycled aggregates and powdered fine recycled aggregate [46]. 

The interfacial zone (Figure 7B), which mainly consists of a large densified layer of hydrates, is similar to that observed by [46]. Beyond 30% replacement of MCRA, the amount and distribution of CSH is insufficient. Thus, the increased replacement of the MCRA results in a large amount of porosity and reduced strength of concrete, which can lower the durability properties that can be seen in Mix 3 at 28 days in Figure 7F. CSH gel decalcifies into silica gel, therefore deteriorating the performance of concrete, which can be seen in Figure 7D at 28 days. The increasing amount of silica gel may produce cracks and loss of durability and mechanical strength in concrete structures. In the case of 100% replacement, the SEM images show more nonhydrated particles due to the incomplete reaction caused by the smaller surface area of the mixture, resulting in a less compact microstructure and strength. Therefore, the increased percentage of recycled content results in inferior properties when compared to the natural concrete.

**Figure 8:**
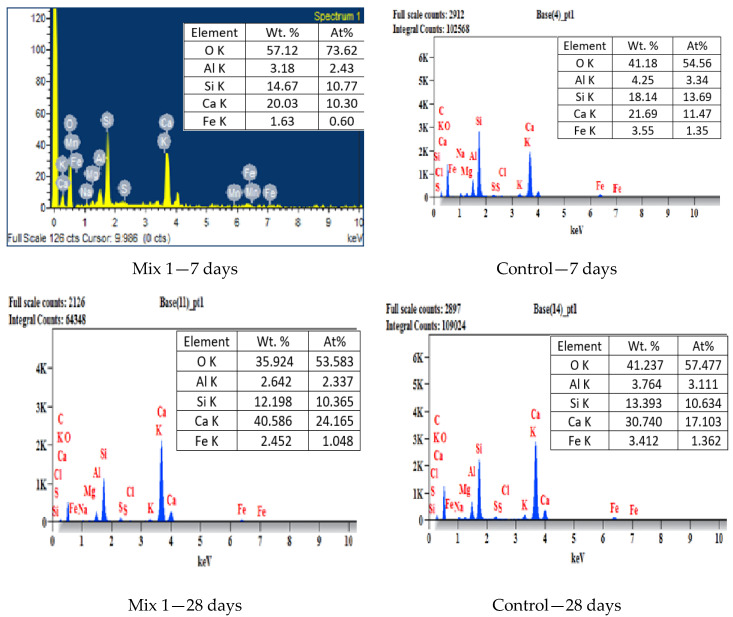
EDS analysis of optimum and control mix at 7 and 28 days.

The energy-dispersive X-ray spectroscopy shows the high content of major peaks of Ca, Si, and O, which indicates the presence of calcium silicate hydrates (CSH) in hardened concrete. The high calcium content shown in Figure 8C indicates the presence of a monolithic amorphous CSH similar to the results reported by [47]. The Ca:Si ratio of the optimum and control mix were calculated from the values shown in Figure 8. At 7 days of curing, the Ca:Si ratio is 0.96 and 0.83 for the optimum and control mix, respectively. After a curing time of 28 days, the results indicate that the Ca:Si ratio of the optimum mix is 2.33 when the control mix results in 1.61. Though the atomic Ca:Si ratio of the optimum mix is slightly higher than that of the control mix used in this study, the results are very similar to those obtained by [48]. The conversion of calcium hydroxide to additional inclusion of CSH is the primary factor for reducing the Ca:Si ratio of CSH. The optimum mix shows a lower alumina content and similar silica content compared to the control mix. Thus, the optimum mix with recycled aggregate has enhanced strength characteristics. 

### 3.3. X-ray Diffraction

The diffractogram was obtained using CuKα radiation (30 mA and 45 kV), scanning between 10.0100 and 89.1500 (°2Th.), as shown in Table 8. The development of mineral phases formed was assessed after a curing time of 7 and 28 by the XRD technique.

#### 3.3.1. Hydrated Samples at 7 Days

**Figure 9:**
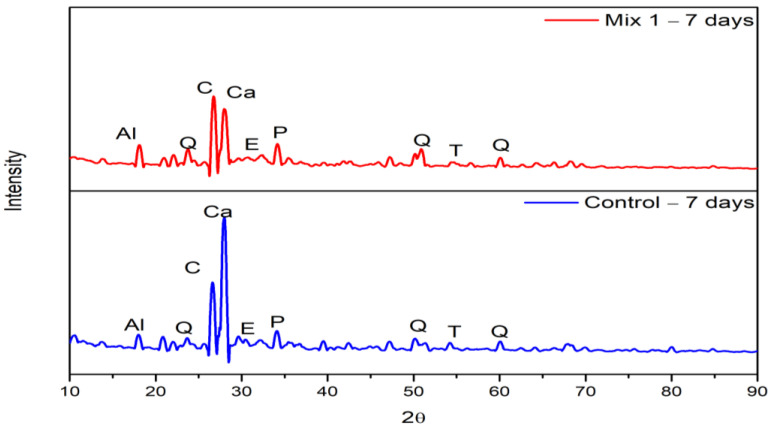
XRD of 7-day hydrated samples (Mix 1 and Control).

The concrete specimen’s X-ray Diffraction (XRD) patterns after seven days of curing are displayed in Figure 9. The control and optimum mix with 30% mixed coarse recycled aggregate have similar patterns. The “X’PERT HIGH SCORE v3.0” software, a commonly used program for the study of the XRD findings, was utilized to analyze the results. This software compares the provided pattern with the reference peaks in the ICDD database and gives the relative intensity of each phase (CSH (C)—PDF Number:33–0306; SiO_2_ (Q)—PDF Number: 46-1045; Ca(OH)_2_ (P)—PDF Number: 44-1481; CaCO_3_ (Ca)—PDF Number: 83-0577), as shown in Table 9. The amount of CSH, CaCO_3_, Ca(OH)_2_, and Al_2_O_3_·3H_2_O are observed at 26.7163°, 28.0422°, 34.1573°, and 18.1007°, respectively, in the optimum mix. Similarly, the amount of CSH, CaCO_3_, Ca (OH)_2_, and Al_2_O_3_·3H_2_O are observed at 26.6157°, 27.9707°, 34.0779°, and 18.0160°, respectively, in the control mix. Likewise, similar results were found by [49] with the formation of CSH, CaCO_3_, and Ca(OH)_2_ compounds at 29.406°, 27.233°, and 34.102°, respectively. The higher counts of CSH in the optimum mix indicates that the strength improvement is higher than that of the control mix, as shown in Table 9. As reported by [50], as the ettringite is a product of an expansive reaction, additional ettringite formation will hasten the deterioration of recycled aggregate concrete, which is not considerably present in the optimum mixes employed in the current study.

#### 3.3.2. Hydrated Samples at 28 Days

**Figure 10:**
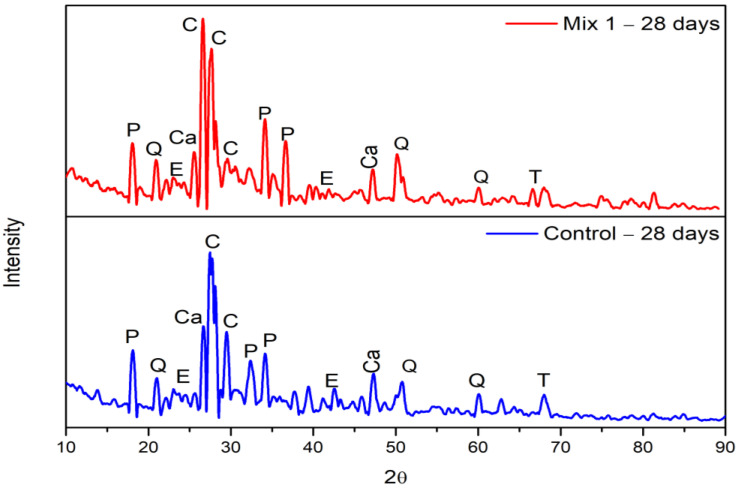
XRD of 28-day hydrated samples (Mix 1 and control).

The optimum and control mix’s XRD patterns after 28 days of curing are shown in Figure 10. The control and optimum mix have similar patterns. The “X’PERT HIGH SCORE” software compares the provided pattern with the reference peaks in the ICDD database and gives the relative intensity of each phase, as shown in Table 10. While seeing the results obtained from the software and patterns, the phase of Ca(OH)_2_ is observed at 18° and 34°, which is similar to the observation reported by [51]. It is also observed that the relative peak intensities of portlandite of the optimum mix slightly increase compared with the result obtained for the control specimen. The highest peak of CSH observed in the 2θ value at 27° is higher than that of the control mix. This result is similar to the results obtained by [52]. The peaks of calcite crystals are found with the highest intensity at 26.60° for both the control and optimum mix, which is similar to the results observed by [53]. Overall, it is observed that the relative peak intensities of all the crystalline phases in the optimum mix are higher than the peaks obtained in the control specimen, as shown in Table 10.

### 3.4. Fourier Transform Infrared Spectroscopy

#### 3.4.1. Hydrated Samples at 7 Days

**Figure 11:**
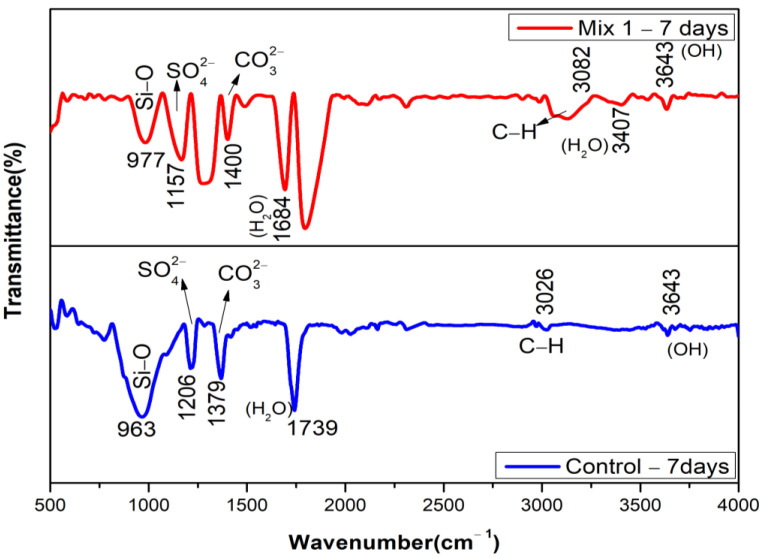
FTIR of 7-day hydrated samples (Mix 1 and Control).

**Figure 12:**
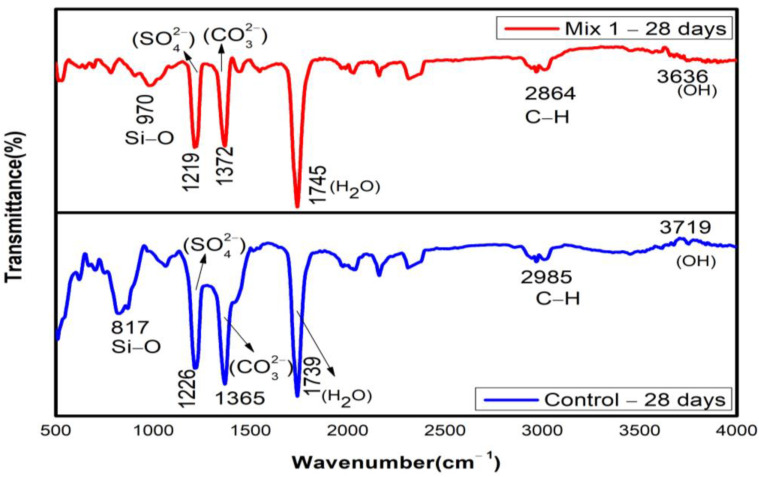
FTIR of 28-day hydrated samples (Mix 1 and Control).

The peaks of bands in the FTIR spectrum at 7 days were identified based on the observation by [54]. The band of Si-O-Si in the control mix exhibits a stretching vibration at a wavenumber of 962 cm^−1^. The replacement of 30% MCRA shifts the Si-O-Si absorption peak toward a higher wavenumber of 978 cm^−1^ in comparison to the control mix. This shift in wavenumber indicates that the silicon phase in the recycled aggregate changes the Ca:Si ratio in CSH. This could be the reason for the increased compressive strength with the addition of 30% MCRA. The peak at 3643 cm^−1^ for both mixtures indicates the presence of portlandite (Ca(OH)_2_), which is revealed by FT-IR spectroscopy. This peak is likely to subside during the period of the exposure, as shown in Figure 11 and Figure 12. The vibration bands at 1684 cm^−1^ and 3407 cm^−1^ for the optimum mix indicate that the O-H group is contributed by CSH, but in the control mix, the O-H group is contributed only at 1739 cm^−1^. The strong vibration bands at 977 cm^−1^ and 963 cm^−1^ for the optimum and control mix, respectively, signify the antisymmetric stretching associated with the Si-O group provided by CSH. The bands at 1379 cm^−1^ in the control mix and 1400 cm^−1^ in the optimum mix correspond to the antisymmetric stretching of CO_3_^2−^. The peak at 1157 and 1206 cm^−1^ associated with SO_4_^2−^ is detected in the control and optimum mix, respectively, which corresponds to the findings by previous research [55] where the typical sulphate absorption bands were usually present between 1100 and 1200 cm^−1^ of wavelength because of the v_3_ vibration in SO_4_^2−^. In the current study, there is no sign of AlO_6_ at 850 cm^−1^, which further reduces the chances of the deterioration of concrete.

#### 3.4.2. Hydrated Samples at 28 Days

The peaks of bands in the FTIR spectrum for both the optimum and control mix at 28 days of hydration are shown in Figure 12. The graph reveals the bond of Si-O located around 817 and 970 cm^−1^ for the optimum and control mix, respectively, where it was observed between 900 and 1100 cm^−1^ in the study conducted by previous researchers. Most likely, the small peaks between 600 and 800 cm^−1^ are brought on by Si-O-Si bonds that come from the original features of the MCRA. It is observed that the intense band at 970 cm^−1^ is due to the Si-O asymmetric stretching vibration of the CSH. The vibration bands obtained at 1649 cm^−1^ and 3432 cm^−1^ for the control mix and 1683 cm^−1^ and 3436 cm^−1^ for the optimum mix represent the antisymmetric stretching associated with the OH- group generated by portlandites. The bands of carbonates (CO_3_^2−^), CSH, and free silica (O-Si-O) are found to be less than the wavelength of 2000 cm^−1^. The presence of the CO_3_^2−^ band at 1365 cm^−1^ for the control mix and 1372 cm^−1^ for the optimum mix are found to be approximately similar to each other. The existence of absorption bands between 2960 and 2850 cm^−1^ that increases the compressive strength of concrete is a sign that stearic acid is present [56]. The considerable C-S-H gel content in both mixes, which is the cause of the strength improvement, is confirmed by FTIR analysis.

## 4. Conclusions

Based on the research findings, the mixed coarse recycled aggregates at 30% replacement can be utilized as an alternative to conventional aggregate to produce recycled aggregate concrete for structural applications. This research can increase the waste utilization in the form of coarse recycled aggregates. Further, the methods for enhancing the mechanical properties can be achieved by the addition of supplementary cementitious materials or various treatment methods, thus contributing to better performance and greater sustainability in construction. This will facilitate the wider use of mixed coarse recycled aggregate in the production of structural concrete as follows.

- Coarse recycled aggregate satisfied the physical parameters specified in IS 383:2016 for the production of concrete.
- The incorporation of coarse recycled aggregates in concrete showed a slump value around 75 ± 20 mm.
- The fresh concrete properties of density with 100% recycled aggregate concrete slightly decreased at a percentage of 2.33%. This behavior was the same for the concrete in fresh and hardened stages.
- The concrete with 30% replacement of MCRA showed increases of 11.01, 6.98, 6.19, and 14.23% at 7, 28, 56, and 90 days, respectively, in comparison with the control mix. Similarly, concrete with 30% replacement of MCRA showed improved split tensile and flexural strengths by about 2.92 and 6.26%, respectively.
- The ultrasonic pulse velocity (UPV) was observed as 4495 m/s for 30% of MCRA, which is recommended as excellent by IS 516 (Part 5/sec1): 2018.
- At 28 days, concrete with 30% incorporation of MCRA and the control mix had rebound numbers of 32 and 36, respectively.
- Scanning Electron Microscopy (SEM) investigations revealed a denser microstructure of recycled aggregate concrete at 30% replacement.
- The mineralogical characterization by X-ray diffraction revealed that the phases found in the recycled aggregates were calcium silicate hydrate, quartz, portlandite, and calcite in the required intensity.
- Due to silicate polymerization and the development of calcium silicate hydrate gel, the primary peak at 817 cm^−1^ changed to a higher frequency 970 cm^−1^ for the optimum mix as hydration advanced.

## Figures and Tables

**Table 1:** Physical and mechanical properties of the aggregates.

SI.No	Properties	NCA		RCA	
		20 mm	12.5 mm	20 mm	12.5 mm
1	Loose air-dried bulk density (kg/m^3^)	1475	1453	1319	1186
2	Bulk density (Rodded) (kg/m^3^)	1668	1642	1452	1381
3	Specific gravity	2.71	2.68	2.3	2.41
4	Water absorption (24 h) %	0.5	0.67	2.3	1.95
5	Aggregate crushing value %	-	16.07	-	31.6
6	Aggregate impact value %	-	13.3	-	27.9

**Table 2:** Water absorption data from literature.

SI. No	Reference	Water Absorption % after 24 h and Size of Aggregates		Source of RA
		CMRA	FMRA	
1	[17]	6.28 (6–12 mm)	5.39 (0–6 mm)	RAPLASA C&DW management plant in Cáceres (Spain).
2	[4]	6.12 (12 mm max)	4.08 (7 mm max)	locally in South Australia.
3	[7]	8.5 (9.5–31.5 mm)	21 (0–9.5 mm)	Hangzhou qianjiang new city municipal garden Construction Co., Ltd.
4	[34]	8.7 (18–32 mm) MRA-1	-	Zhoushan jinke resources recycling Co., Ltd. in Zhoushan, China.
		9.1 (10–18 mm) MRA-2	-	
		13.2 (0–18 mm) MRA-3	-	
5	[14]	9.1 (4–22 mm)	-	CDW recycling plant at Lisbon (Portugal).
7	[19]	9.1 (0–25 mm)	-	
8	[35]		6.09 (<4.75 mm)	CDW recycling plant n São José do Mipibú Brazil.
9	[18]	5.27 (22/12 mm) 6.28 (12/6 mm)	-	C&DW plant in Extremadura, western Spain.
10	[36]	17.82	16	construction and demolition waste treatment plant, Spain.
11	[37]	-	5.23 (<4.75 mm)	Collected from Porto Alegre (RS, Brazil).
12	[8]	4.49 (<20 mm)	-	C&DW management and processing plant in Glasgow, Scotland.

**Table 3:** Composition of the concretes designed.

Materials	Mix (kg/m^3^)			
	Control	M1	M2	M3
Cement	380	380	380	380
NCA (20 mm)	697	488	279	-
NCA (12 mm)	460	322	184	-
MCRA (20 mm)	-	188	374	624
MCRA (12 mm)	-	122	243	406
MSAND	725	725	725	725
WATER	160	160	160	160

**Table 4:** Concrete properties in a fresh state.

Mix	Slump Value (mm)	Fresh Density (kg/m^3^)	Temperature (°C)
Control	75	2416	28
M1	90	2463.3	27
M2	80	2473	27
M3	90	2386.67	27

**Table 6:** Split tensile and flexural strength of concrete at 28 days.

SI.NO	Mix ID	Split Tensile Strength, *f_c_* (MPa)	$∆f_{c}\;(\%)\;♣$	Flexural Strength, *f_b_* (MPa)	$∆f_{b}\;(\%)\;♣$
1	M1	2.82	+2.92	5.09	+6.26
2	M2	2.29	−16.42	3.65	−23.80
3	M3	2.21	−19.00	2.52	−47.39
4	Control	2.74	-	4.79	-

♣ strength variation compared to control mix.

**Table 7:** Results of UPV and rebound hammer test.

Si. No	Mix ID	Ultrasonic Pulse Velocity (UPV) @ 28 Days			Rebound Value
		Velocity (m/s)	Time Taken (microseconds)	Concrete Quality	
1	M1	4495	33.37	Excellent	32
2	Control	4655	32.22	Excellent	36

**Table 8:** X-ray diffraction data collection.

Equipment	Manufacturer	PANlytical, Bruker, Rigaku
	Model	XPERT-PRO
Diffractometer Geometry	Measurement setup	Bragg–Brentano, transmission; θ–θ, θ–2θ
X-ray Source	Goniometer Radius (mm) X-ray radiation Generator Settings	240 Anode Material Cu K-Alpha1 (Å): 1.54060 30 mA, 45 kV
Scan parameters	Size (°2Th.) Scan Time (s) Type Scan Axis	0.0200 1.0000 Continuous Gonio

**Table 9:** Peak list of 7-day hydrated concrete sample.

SI.No	Mix	Phase	Pos. (°2Th.)	Height (cts)	FWHMLeft (°2Th.)	d-Spacing (Å)	Rel. Int. (%)
1	Mix 1	CSH	26.7163	398.17	0.1181	3.33409	100.00
		CaCO_3_	28.0422	196.89	0.1378	3.17939	49.45
		Ca(OH)_2_	34.1573	70.57	0.1574	2.62288	17.72
		Al_2_O_3_·3H_2_O	18.1007	95.76	0.1181	4.89693	24.05
		SiO_2_	60.0231	41.02	0.0720	1.54006	10.30
			50.8314	85.93	0.0720	1.79481	21.58
			23.7228	70.35	0.0590	3.74760	17.67
2	Control	CSH	26.6157	308.31	0.1181	3.34646	61.22
		CaCO_3_	27.9707	503.58	0.1181	3.18735	100.00
		Ca(OH)_2_	34.0779	52.15	0.1574	2.62881	10.36
		Al_2_O_3_·3H_2_O	18.0160	49.86	0.1181	4.91977	9.90
		SiO_2_	59.9690	27.89	0.0720	1.54132	5.54
			50.1300	39.33	0.0720	1.81826	7.81
			23.5069	26.79	0.1968	3.78152	5.32

**Table 10:** Peak list of 28-day hydrated concrete sample.

SI. No	Mix	Phase	Pos. (°2Th.)	Height (cts)	FWHMLeft (°2Th.)	d-Spacing (Å)	Rel. Int. (%)
1	Mix 1	CSH	27.4555	198.56	0.0787	3.24598	100.00
		CaCO_3_	26.6737	185.10	0.0787	3.33932	93.22
			47.1950	25.50	0.1968	31.92426	49.45
		Ca(OH)_2_	18.0747	89.26	0.0787	4.90393	44.95
			34.0967	79.89	0.0787	2.62741	40.24
			36.5634	96.23	0.0590	2.45561	48.46
		Quartz	50.1765	66.24	0.0720	1.81668	33.36
			20.8716	52.68	0.0787	4.25266	26.53
2	Control	CSH	27.4368	280.59	0.1181	3.24815	100.00
		CaCO_3_	26.7069	101.37	0.0787	3.33525	36.13
			47.1204	32.29	0.1574	1.92713	11.51
		Ca(OH)_2_	18.0999	88.34	0.0984	4.89713	31.48
			34.1388	43.47	0.1968	2.62426	15.49
			37.7077	35.93	0.0590	2.38368	12.81
		Quartz	50.6358	18.08	0.2362	1.80128	6.44
			21.0258	33.66	0.1968	4.22182	12.00

## Data Availability

Not required.
